# Education in Academic Emergency Medicine During the COVID-19 Pandemic – Our Experience From an Ongoing Crisis

**DOI:** 10.3389/fpubh.2020.592503

**Published:** 2020-10-30

**Authors:** Matthias Mueller, Christoph Schriefl, Michael Holzer, Martin Roeggla, Anton N. Laggner, Florian Ettl

**Affiliations:** Department of Emergency Medicine, Medical University of Vienna, Vienna, Austria

**Keywords:** medical education, pandemic (COVID-19), emergency medicine, infectious disease, COVID

## Abstract

**Background:** The COVID-19 pandemic has resulted in the suspension of the entire teaching program at the Medical University of Vienna till the end of the summer semester. As the department that is responsible for emergency medicine teaching, we adapted the program to continue the courses and maintain the learning progress. Our objective is to evaluate the number of courses conducted and report the methods used.

**Methods:** Teaching was measured as credit hours per week (CHW) in accordance with the university's prospectus. One CHW represents 15 academic hours (45 min) in one semester. Webinars were conducted using the CISCO Webex Events®, Webex Training, and ZOOM®. The Moodle® was utilized for resuscitation courses.

**Results:** Courses and clerkships equivalent to 80.2 out of 101.4 CHW (79.1%) could be held during the ongoing crisis in the summer semester. Courses in the winter semester were all completed. In the human medicine curriculum, 73.7 out of 94.9 CHW (77.7%) could be conducted. In the case of emergency lectures for the dentistry curriculum, all courses were conducted through webinars (6.5 CHW, 100%). After calculating the exact number of students in each class, it has been determined that courses and clerkships equivalent to 78.7% could be conducted.

**Conclusion:** Despite the challenge of preparing for the treatment of numerous patients during the ongoing pandemic, we could shoulder a majority of our teaching responsibilities. Although sufficient skill training could not be imparted under these circumstances, we could provide sufficient theoretical knowledge to allow students to continue studies.

## Introduction

On February 25, 2020, the first cases of COVID-19 were diagnosed in Austria. Subsequently, the number of new confirmed cases began to double over a span of 2 to 3 days (1). As a part of the effort to “flatten the curve” and minimize transmission of the virus, the entire teaching program of the Medical University of Vienna was suspended with immediate effect from March 10, 2020. This decision also affected the clinical training for students in the last two academic years, although those in the last year could continue their clinical internship year (the so called “clinical practical year”) voluntarily. It was determined that teaching should be conducted through distance learning programs with webinars or other online teaching media at the discretion of the responsible departments. This change was first estimated to last for 2 weeks but gradually extended till the end of the summer semester.

As the Department of Emergency Medicine, we expected numerous patients with COVID-19. The high number of infected and deceased healthcare workers reported in China and Italy caused disturbances and led to anxiety among the medical staff (2, 3). Following the soaring number of critically ill patients in these countries, we utilized this period to prepare for the worst.

Additionally, we began to plan ways to implement distance learning for our courses. Our department was the first chair in emergency medicine in German-speaking countries and is responsible for the planning and conducting of courses in all sections of human medicine as well as dentistry. At the point at which the teaching program had to be suspended, several courses were ongoing and some lessons had been held already.

We had to design a program that fulfills both the requirements of protecting students and staff members through physical distancing and continuing teaching whenever possible. As stated, this had to be achieved alongside ensuring availability for the suspected number of COVID-19 patients.

To achieve this, we decided to stop all elective subjects and concentrate on the mandatory elements of the curriculum despite the limitations.

This paper provides an overview of the ways in which our department handled teaching during these exceptional circumstances and has managed to continue teaching a relevant part of the courses.

## Materials and Methods

### Setting

The Medical University of Vienna is the largest medical university in German-speaking countries, having about 8,000 medical students. The Department of Emergency Medicine (ED) and all other clinical departments are located in the General Hospital of Vienna, a tertiary care center with 1,728 beds.

Our department is divided into an outpatient ward, a resuscitation bay with all intensive care facilities, and an intermediate care station for 10 patients. Clinical and scientific attention is focused on patients with cardiac arrests, myocardial infarction, arrhythmias, strokes, and other life-threatening conditions.

Being an academic teaching center, education is one of the core tasks. Despite the low staffing levels and large clinical volume, we fulfill a notable part of the university's teaching responsibilities (3.14%). In terms of per capita ranking, we rank second in all clinical and preclinical departments.

### Emergency Medicine in the Medical Curriculum in Vienna

The ED is responsible for training students in medicine and dentistry degree programs. The training includes first aid, resuscitation, and critical care medicine. This ranges from first aid training in the first semester to intermediate and advanced life support (resuscitation training I and II) as well as scenario-based resuscitation training as the students progress.

In addition to the clinical activities, a core team supervised by the head of department is responsible for the organization, implementation, and content creation of the courses and clinical internships. They receive guidelines and instructions from the curriculum management team and are entrusted with further implementation.

In this exceptional situation, a core team for distant learning was formed to plan the changes for this semester and present them for approval to the head of department and the curriculum management team.

### The Clinical Practical Year

In this 16-week course, students work as an integrated part of our staff in the ED under direct supervision for 35 h per week. This is accompanied by exclusive tutorials held by physicians or senior nurses from our team. During the academic year, we oversee three rotations with 10 students. Each student is allocated a mentor who is responsible for developing their technical and non-technical qualifications. Structured feedback is given at the beginning, during the midterm as well as at the completion of the clinical practical year, and through well-evaluated feedback tools— “direct observation of procedural skills” (DOPS) and “mini-clinical evaluation exercise” (mini-CEX) (4, 5).

### Hygiene Protocols During the COVID-19 Pandemic

At our ED, all employees have to wear FFP-2 masks, goggles, double non-sterile gloves, and surgical caps as soon as they enter the treatment rooms. In social areas, they wear FFP-1 or surgical masks and are instructed to maintain a distance from other colleagues. All patients are given surgical masks as soon as they enter the hospital. Prior to aerosol generating procedures and treatment of COVID-19 suspicious cases, long-sleeved gowns, FFP-3 masks, and face shields are added to the standard personal protective equipment (PPE). These precautions are in accordance with the World Health Organization and the European Center for Disease Prevention and Control (6).

### Software

To enable direct interaction with students, we use the voice over IP-services—Webex Events® (Cisco Systems, Inc., San Jose, CA, United States) and ZOOM® (Zoom Video Communications, Inc., San Jose, CA, United States). Both software tools permit the sharing of slides and communication with students through video-chat.

Furthermore, we use Webex Training® (Cisco Systems, Inc., San Jose, CA, United States) to implement multiple-choice questionnaires during some of the lectures.

For resuscitation courses, we use the learning platform and course management system, Moodle® (M. Dougiamas et al., under GNU General Public License, http://www.moodle.org).

### Credit Hours per Week

The Medical University of Vienna monitors their whole teaching in the form of credit hours per week. The average semester lasts for 15 weeks. Therefore, a weekly course with a duration of one academic hour (45 min) is calculated as one credit hour per week (CHW). For our computation, we collected the official numbers from the prospectus of the university; if they were not available, we calculated the CHW using the aforementioned formula.

Statistical calculations and data management were performed using Microsoft Excel for MAC V16.3 (Microsoft Corporation, Redmond, WA, United States).

### Ethical Aspects

An exemption was obtained by the Ethical Committee of the Medical University of Vienna, as no personal data have been utilized.

## Results

Overall, courses and clerkships equivalent to 80.2 out of 101.4 CHW (79.1%) were held during the ongoing crisis in the summer semester. Courses in the winter semester were completed as per the regular schedule. In the human medicine curriculum, we could conduct 73.7 out of 94.9 CHW (77.7%). In the emergency lectures for the dentistry curriculum, we could conduct all courses through webinars (6.5 CHW, 100%). After calculating the exact number of students in each course during this semester, it was determined that courses and clerkships equivalent to 78.7% (76.3% in human medicine and 100% in the dentistry curriculum) could be conducted (Table 1).

**Table 1:** Conducted courses during summer semester 2020.

**Crude data from the university's prospectus**			**After adjustment for the exact number of students in each course**	
	**planned**	**conducted**	**planned**	**conducted**
Human medicine in CHW (%)	94.9	73.7 (77.7)	1120.5	855.0 (76.3)
Dentistry in CHW (%)	6.5	6.5 (100.0)	126.5	126.5 (100)
Total in CHW (%)	101.4	80.2 (79.1)	1247.0	981.5 (78.7)

*CHW: credit hours per week*.

The courses that are the responsibility of the ED, along with their current status, are listed in Tables 2,3.

**Table 2:** Overview of the courses held during summer semester 2020—human medicine curriculum–mandatory.

**Subject**	**Semester**	**Status**
First aid	1	Completed in winter semester
Resuscitation training I	5	Completed in winter semester
Resuscitation training II	8	Converted to interactive moodle course
Objective Structured Clinical Examination and preceding training	8	Suspended as per the dean
Clinical training in emergency medicine (5 week rotation)	9–10	Suspended as per the dean
Clinical practical year, emergency medicine trimester (16 week rotation)	11–12	Frequent webinars, special training in hygiene aspects, modification of roster to reduce the number of simultaneously attending students and to avoid overlap with different physician-teams on duty

**Table 3:** Overview of the courses held during summer semester 2020—dentistry curriculum—mandatory.

**Subject**	**Semester**	**Status**
First aid	1	Completed in winter semester
Emergency medicine lectures	9–10	Held as webinar
Emergency medicine training	9–10	Held as webinar
Refresher in emergency medicine	11–12	Held as webinar

### Resuscitation Training II

Over the winter semester, we designed an interactive Moodle® course with short videos on ways to perform cardiopulmonary resuscitation and rhythm checks correctly, as well as use of various airway management devices. Furthermore, we introduced excerpts from the current resuscitation guidelines followed by mandatory questions, interactive video clips, and drag and drop tests. Although this course was primarily designed to help students to be well-prepared for the training sessions, it is now also used as a replacement for traditional courses in an adapted form.

### Clinical Practical Year

A new group of students arrived 1 week after the teaching suspension. Due to the multiple ongoing changes in the ED (e.g., establishment of a hygiene-lock between social areas and treatment rooms, hygiene training for our staff, pandemic-related spatial concept), lessons were conducted through daily webinars in the first 3 weeks. Using this method, we trained students on our department-specific processes, gave lectures on frequent reasons for consultation (e.g., chest pain), suggested techniques (e.g., emergency sonography, extracorporeal life support), and encouraged students to present emergency-related cases from former clerkships.

While ensuring the safety of students in the ward, we trained two students at a time to correctly handle PPE material. Only students without preconditions were allowed to attend these sessions. We also clarified that attendance is strictly voluntary, as specified by the dean.

We rearranged the roster to a more flexible system to reduce the number of students attending sessions simultaneously and to avoid overlaps between different physician teams on duty. Three “COVID-mentors” were tasked with providing continuous mentorship, arranging more webinars, and offering support during mentally challenging situations.

### Dentistry Curriculum

All courses were conducted through webinars with the option of direct student–teacher interaction. For practical aspects in the courses, flexible solutions were found (e.g., recovery position training with people living in the same household, chest compressions on pillows). Procedures that require equipment were demonstrated using video clips.

## Discussion

This paper elaborates on the fact that even during the COVID-19 pandemic, emergency medicine education is feasible both through distance and face-to-face learning. We managed to conduct 79.1% of the courses (78.7% after correction for the exact number of students) in the summer semester. The experiences garnered during this global crisis could help improve teaching through webinars and in clinical clerkships in the case of future epidemics or pandemics. To the best of our knowledge, no data on the teaching of medical students during the COVID-19 pandemic in the field of emergency medicine are available to date.

### Students' Role During COVID-19

Medical schools in various countries have been closed to mitigate the spread of the virus (7, 8). One of the central teaching aims in emergency medicine is self-protection and personal security for the aides. The risk of infection with COVID-19 is high, especially during aerosol generating procedures such as airway management, cardiopulmonary resuscitation, or non-invasive ventilation, which are common in EDs. Furthermore, it is quite impossible to detect all infected patients at pre-triage, resulting in a high contagion risk while handling the patient. Therefore, an ED is probably the most inappropriate place for clerkships during a pandemic.

Concurrently, students' motivation to help combat the upcoming crisis was high. We received several proposals from former or scientifically active students from our ED to support us with the pending duties, at a time when we had not seen a single COVID-19 patient. Some of them are also trained as paramedics and familiar with our routines, and therefore they might be helpful if the ED is strained.

However, COVID-19 is highly transmissible (9). The World Health Organization stated that 3.7% of laboratory-confirmed cases in China were healthcare workers (2). In Italy, our bordering country, 20% of healthcare workers dealing with COVID-19 patients have also been infected (3).

Accordingly, we discussed whether it would be responsible to allow students to enter the ED under these circumstances. Due to a global shortage of PPE and a high chance of virus transmission, we decided not to permit the attendance of voluntary students or further clerkships. The same decision was made in the USA and the UK (7, 10), even though the act of fast-tracking final year students to get them registered as doctors quickly is under debate in both countries (11, 12). For the 10 students in the clinical practical year, we made an exception because of certain considerations. Most of the healthcare workers have been infected in a few specific but frequent situations, including unprotected contact; in particular, aerosol generating procedures and self-contamination when removing PPE bear an increased risk (13). We therefore ensured that our students are properly trained in the use of PPE. They are not allowed to perform aerosol producing procedures and are further instructed to maintain sufficient distance in case of possible aerosolization. Along with regular debriefing of organizational, social, and medical issues through video conferences with the COVID-19 mentors, close-meshed support is guaranteed, and emerging problems can be intercepted at early stages. In our opinion, given the small number of students, a clinical clerkship is justifiable.

### Distance Learning

Distance or blended learning has been widely implemented in medical education over the last few years (14). Fortunately, this gave us the opportunity to adapt to the new situation quickly.

Before the crisis, our university used Moodle®, a virtual microscope, and several video tutorials on ways to perform clinical examinations or procedures. Additionally, the Medical University of Vienna streams the “Grand Rounds Lectures” to students in the Erasmus exchange program who are therefore absent during the fifth year.

Although video conferencing and messaging were not widely used previously, these were quickly adopted after shutdown and seem to be appropriate tools for lectures and seminars. The same adaptions for the COVID-19 pandemic have been reported by Chick et al., Moszkowicz et al., and Zhou et al. (15–17).

In the normal learning environment, a primary aspect of our courses is practical skill training (e.g., cardiopulmonary resuscitation, airway management, assessment of emergency patients). Distance learning can never be an adequate substitute for hands-on teaching with proper equipment. Nevertheless, in the absence of alternatives, we have attempted to adapt to the best of our abilities. All practical aspects are taught several times over the course; therefore, we can ensure that all students achieve this learning objective, although special focus on correct execution will be needed in future courses.

### Strengths and Limitations

To our knowledge, this is the first article on the realization of educational aspects for students of an academic ED during the COVID-19 pandemic. These data might be useful for other institutions in the fields of emergency, intensive care, or respiratory care medicine that strive to continue their courses. Moreover, we believe that some of the evolved techniques have the potential to be implemented in our teaching methods during times with lower workload to help our students achieve the highest possible level of learning success. Additional work for the implementation of our online elements has been performed on a voluntary basis by the colleagues who were most involved in teaching the courses. This helped in saving costs and protecting staff from excessive demands.

The study has certain limitations that need to be discussed. With CHW, we are only able to report the amount of teaching undertaken. As this is an ongoing crisis, we are unable to evaluate our teaching with reference to achieving learning objectives or learning satisfaction at this point. Although we are able to pursue the same staff–student ratio as before the crisis with the methods used, such data can only be surveyed after regular sessions begin. It is therefore necessary to examine the impact of COVID-19 on medical education globally at a later point in time. Further, it is not possible to compare our efforts with other institutions at this time due to a lack of data. At a later timepoint, a structured survey on how this situation was handled in other departments would be interesting.

### Lessons Learned and Outlook

Informal feedback from students, collated through Moodle, through mail, or personally, has been consistently positive. The students were mostly grateful for our quick reaction in this exceptional situation and the opportunity to achieve the majority of their learning goals despite the circumstances. Furthermore, students appreciated the flexibility that these new teaching methods offered. Clinical colleagues also expressed a predominantly positive opinion about the students' cooperation, the adapted training, and their clinical activities under the given circumstances.

After the initial and temporary solutions implemented during the spring session, the Medical University of Vienna and its departments used the summer months to redesign the curricular elements to meet the “new normality” in the upcoming winter semester. While in-person teaching was suspended initially, a more differentiated approach will be put into place for the next semester. Wherever suitable, teaching should be held virtually (e.g., streamed or recorded lectures). In contrast, hands-on training has been recognized as an indispensable component in medical education and must be offered. The departments were required to develop suitable concepts within their responsibilities and present them to the accrediting body. For the purpose of training, the following measures will be implemented. Time schedules and group sizes are rearranged to reduce the simultaneous presence of a high number of students. To ensure sufficient distance between participants, bigger rooms usually used for lectures and seminars will be used for skill training. The use of surgical masks and periodic hand and surface disinfection is mandatory. The lecture hall center will be strictly separated from the clinical area of the university hospital, and a one-way system at the entrances and exits will be established. Support staff will be monitoring the flow of students. Currently, the first academic institution in Austria has started using antigen quick tests for students. With broader availability and low prices, this might be another strategy to reduce the hazard of infection during training. With these measures, it is possible to provide both safe and sufficient teaching over the next semesters.

A further goal is an evaluation of the measures taken and their impact on the students' theoretical and practical skills after the coming semesters. This might be facilitated by an “objective structured clinical examination” (OSCE).

## Conclusion

Despite the challenge of preparing to treat numerous patients during an ongoing pandemic, we could maintain the majority of our teaching responsibilities. Although sufficient skill training cannot be achieved under these circumstances, we could provide sufficient theoretical background to allow students to continue their studies. We hope that our experiences will inspire other institutions to maintain their educational duties during the COVID-19 pandemic and, in certain aspects, act as a model for blended learning after normal university life resumes.

## Data Availability Statement

The raw data supporting the conclusions of this article will be made available by the authors, without undue reservation.

## Ethics Statement

Ethical review and approval was not required for the study on human participants in accordance with the local legislation and institutional requirements. Written informed consent from the patients/participants was not required to participate in this study in accordance with the national legislation and the institutional requirements.

## Author Contributions

MM, CS, and FE contributed to conception and design of the study. MH provided data and experiences. MM performed the statistical analysis and wrote the first draft of the manuscript. All authors contributed to manuscript revision, read, and approved the submitted version.

## Conflict of Interest

The authors declare that the research was conducted in the absence of any commercial or financial relationships that could be construed as a potential conflict of interest.
